# Enhancing patient care in BPPV-related residual dizziness: introducing the CLEAR algorithm to support BPPV-RD recognition and follow-up strategies

**DOI:** 10.3389/fneur.2025.1689617

**Published:** 2026-01-02

**Authors:** Herman Kingma, Leonardo Manzari, Nuri Özgirgin

**Affiliations:** 1 Balance & Dizziness Centre, Department of Otorhinolaryngology, Head & Neck Surgery and Audiology, Aalborg University Hospital, Aalborg, Denmark; 2 Department of Clinical Medicine, Aalborg University, Aalborg, Denmark; 3 Maastricht University Medical Center, Maastricht, Limburg, Netherlands; 4 Vestibology Science, MSA ENT Academy Center, Cassino, Lazio, Italy; 5 Department of Otolaryngology - Head and Neck Surgery, Bayindir Sogutozu Hospital, Ankara, Türkiye

**Keywords:** BPPV, residual dizziness, algorithm, risk factors, vestibular compensation, type 1 BPPV-related residual dizziness, type 2 BPPV-related residual dizziness

## Abstract

Benign paroxysmal positional vertigo (BPPV)-related residual dizziness (RD), a type of dizziness following successful treatment of BPPV, has been increasingly recognized, with a reported prevalence ranging from 23 to 70%. BPPV-related RD is characterized by non-specific dizziness in the absence of positional vertigo and nystagmus. It can be very distressing and lead to substantial impacts on the quality of life and morbidity, especially the risk of falling. This review examines the risk factors and underlying mechanisms contributing to BPPV-related RD, focusing on peripheral and central mechanisms. Based on clinical experience, two subtypes of BPPV-related RD are suggested: type 1, the classic BPPV-related RD occurring after canalith repositioning maneuvers; and type 2, a novel subtype arising after spontaneously resolved BPPV that requires a history of BPPV but not previous confirmation by clinical examination (subjective BPPV). This review introduces a special online algorithm, the Clinician-Led Evaluation for Assessment of Residual dizziness (CLEAR), to help clinicians recognize patients with BPPV-related RD, and reviews follow-up strategies. The aim is to help specialist ear, nose, and throat clinicians and neurologists recognize BPPV-related RD quickly and follow up appropriately to resolve symptoms as quickly as possible.

## Graphical Abstract

**Figure:**
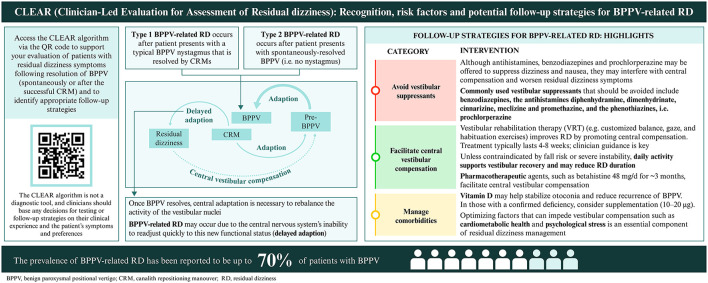
CLEAR (Clinician-Led Evaluation for Assessment of Residual dizziness): Recognition, risk factors and potential follow-up strategies for BPPV-related RD.

## 1 Introduction

Benign paroxysmal positional vertigo (BPPV)-related dizziness (RD) is defined as a sensation of non-specific dizziness in the absence of positional vertigo and typical nystagmus that follows successful treatment with canalith repositioning maneuvers (CRMs) of BPPV (1). BPPV-related RD is being reported increasingly (1). Given the high levels of undiagnosed BPPV at all ages (2), particularly in older individuals (3), it is reasonable to consider that the prevalence of BPPV-related RD is much higher than that currently reported in the literature. In addition, the increase in the reported prevalence is mirrored by an increase in the number of studies investigating the risk factors for RD, especially in patients aged >50 years. BPPV-related RD is likely to be a heterogeneous condition, and this review focuses on how to improve the recognition of patients with a high risk of BPPV-related RD and provide follow-up strategies to reduce or manage this risk according to the underlying etiology.

Patients with BPPV-related RD have reported symptoms, such as continuous or intermittent lightheadedness (4–7), floating sensation (5), head heaviness (8), and imbalance/unsteadiness (4, 7, 8), combined with a clinical history of BPPV resolved after CRMs.

Although BPPV-related RD may be evaluated using scores generated by questionnaires such as the Dizziness Handicap Inventory, Visual Vertigo Analog Scale, and Vertigo Symptom Scale, these have not yet been validated for the diagnosis of BPPV-related RD. Displacement of otoconia from the utriculus into the semicircular canals has been suggested as the primary cause of BPPV (1), and the associated utricular pathology might also play a role in BPPV-related RD. However, objective measures to assess utricular function have not been established yet (1).

Whether dizziness symptoms that remain after a successful CRM for BPPV are present at the time of BPPV diagnosis or whether they are new symptoms that develop after CRM is not known and will be hard to differentiate due to factors that may obscure the distinction between vertigo and dizziness, such as the following:

i) the similarity between dizziness and vertigo symptoms and the often interchangeable use of the terms (although dizziness is a general feeling of being off-balance, whereas vertigo is the feeling that you or your surroundings are moving or spinning),
ii) the vertigo symptoms may overwhelm the dizziness, such that the dizziness is simply absorbed into the vertigo diagnosis, and
iii) patients may have been feeling dizzy before the BPPV but were managing their symptoms and did not report them.

There is clearly an unmet need to improve the definition and diagnosis of BPPV-related RD so that patients can be given appropriate advice or treatment to assist in their recovery.

## 2 Prevalence of BPPV-related RD

**Figure 1:**
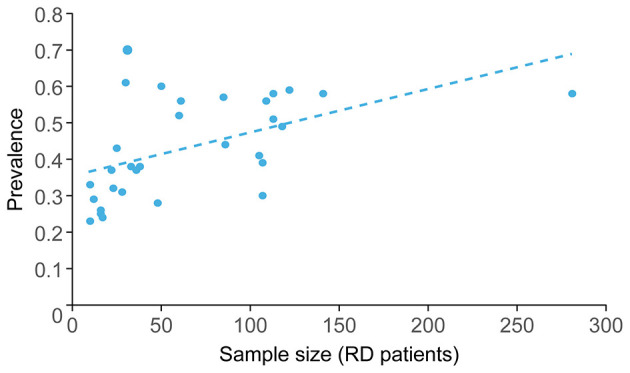
Studies in BPPV-related RD have small cohorts, with a trend toward an association between cohort size and prevalence (9). BPPV, benign paroxysmal positional vertigo; RD, residual dizziness.

**Figure 2:**
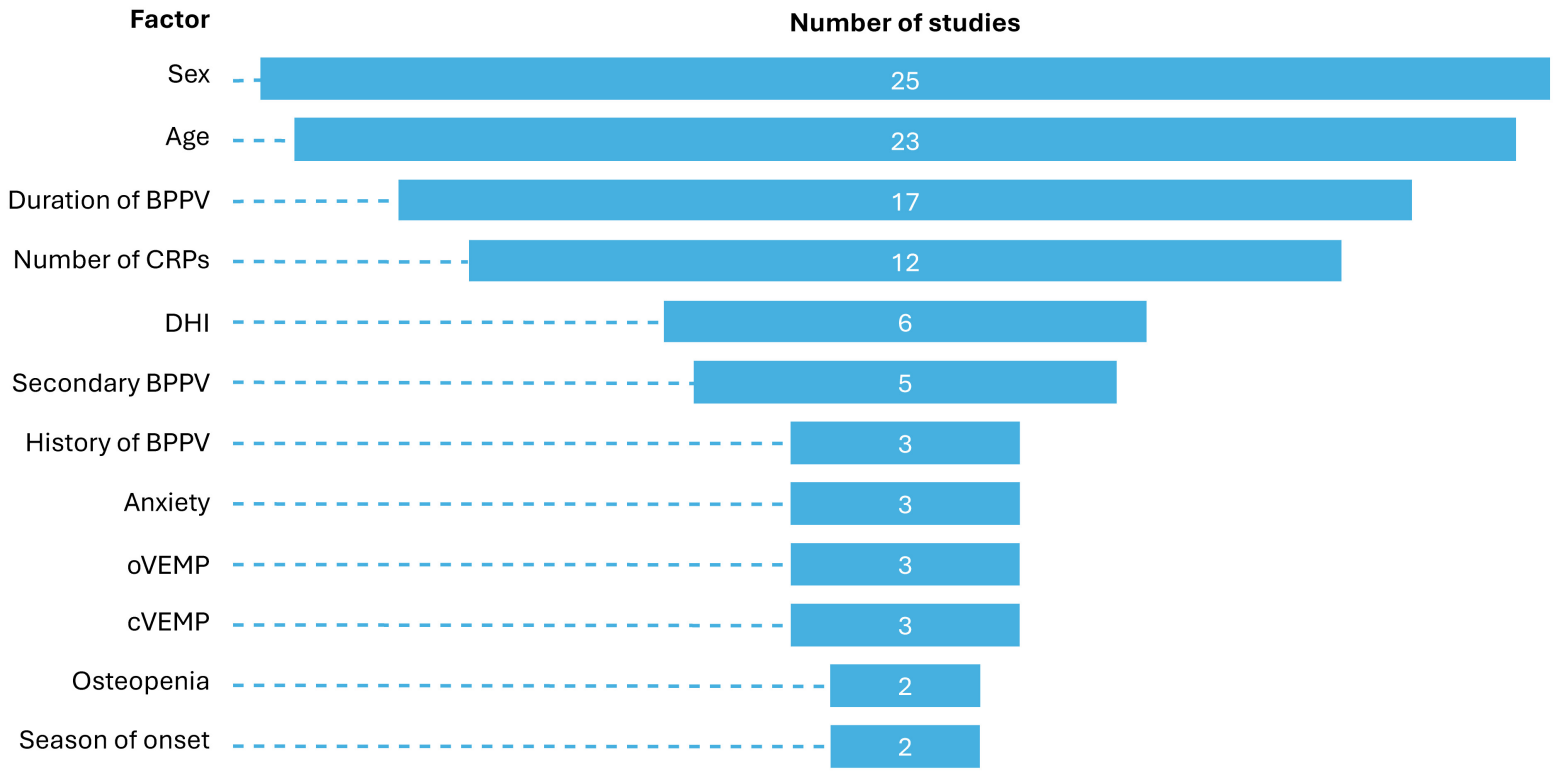
The relatively narrow focus of interest for studies of risk factors for BPPV-related RD (9). BPPV, benign paroxysmal positional vertigo; CRP, canalith repositioning procedure; cVEMP, cervical vestibular evoked myogenic potential; DHI, Dizziness Handicap Inventory; oVEMP, ocular vestibular evoked myogenic potential; RD, residual dizziness.

A systematic review and meta-analysis of risk factors for BPPV-related RD from 31 papers highlighted a wide range of reported prevalence, from 23 to 70% (9). Most of the studies included in the review had small sample sizes, ranging from 20 to 281 patients with BPPV-related RD (17/31 studies included < 50 patients with BPPV-related RD; Figure 1). The two largest cohorts were from >10 years ago, highlighting the limitations of the available data in making accurate statements regarding the prevalence of BPPV-related RD. The disparities in reported prevalence may also reflect the focus of the studies (Figure 2) and/or population differences, as 22 studies were conducted in China.

Positional nystagmus may be considered a common finding amongst healthy individuals without a typical history of BPPV (10). However, this common finding does not increase the incidence of BPPV-related RD (or result in its overdiagnosis), as BPPV-related RD can only be defined when there is a confirmed diagnosis of prior BPPV or a clinical history that has been confirmed by a specialist as highly suggestive of spontaneously resolved BPPV. Furthermore, in patients with a confirmed diagnosis of previous BPPV or a clinical history strongly indicative of spontaneously resolved BPPV as confirmed by a specialist, any remaining nystagmus should not be classified as RD but rather suggests the presence of an otolith that has not yet been repositioned. Accurate diagnosis requires a detailed clinical history to assess whether the patient has no clinical history of BPPV or a clinical history highly suggestive of BPPV.

## 3 Impact of BPPV-related RD

BPPV-related RD symptoms are very distressing to patients, and the ensuing morbidity and poor quality of life (QOL) have been described as formidable and intimidating in a study in India (7). Although this study had relatively few patients reporting BPPV-related RD, those who did reported that they had not regained their sense of stability, indicating that the impact of the BPPV-related RD was worse than the BPPV itself, as they could avoid vertigo by not bending the head into the provoking position but could not avoid the BPPV-related RD symptoms (7). In a meta-analysis of 14 articles, dizziness in older adults was associated with significantly higher odds of any type of future fall and was an independent predictor of future falls (11). Dizziness in the elderly has also been linked to isolation, depression, and reduced self-autonomy and self-control (12). Data from the 2001 to 2004 National Health and Nutrition Examination Surveys in 5,086 US adults aged ≥40 years identified that 35.4% had vestibular dysfunction, as measured by the modified Romberg Test of Standing Balance on Firm and Compliant Support Surfaces, with increased odds based on age and the presence of diabetes mellitus (13). Participants with vestibular dysfunction who reported dizziness had a 12-fold increase in the odds of falling, which is noted as being among the most morbid and costly health conditions affecting older individuals in the US (13). We hypothesize that BPPV-related RD might have a similar impact as the dizziness mentioned above.

Studies on BPPV have reported that ~86% of patients with BPPV will suffer some interrupted daily activities, and, for patients of working age, this includes lost workdays (14). Although BPPV can occur at all ages, there are few, if any, studies on BPPV-related RD in children or young adults, and very few that include adults aged < 50 years. A retrospective analysis of 203 children (mean age, 11.16 ± 3.87, range 1–17 years) who underwent vestibular assessment in a vertigo center in a tertiary hospital over a 3-year period reported that BPPV was the most common diagnosis (49%), which included both primary and secondary BPPV that was associated with other vestibular pathologies (15). Another pediatric study reported that BPPV is a relatively frequent cause of dizziness in children, with common delays in identification and treatment (16). In a study of recurrent dizziness, defined as episodes of dizziness several times a week during the last 6 months, in >40,000 Danish children aged 11–15 years, dizziness was significantly associated with short sleep duration, skipping breakfast, use of alcohol and tobacco, loneliness, low life satisfaction, low self-esteem, exposure to bullying at school, high schoolwork pressure, low school satisfaction, underweight, overweight, poor self-rated health, chronic illness, injuries in the last year, headache, stomach ache, back pain, feeling low, irritability/bad temper, nervousness, difficulties falling asleep, and poor/restless sleep (17). Although this form of dizziness is not related to BPPV, we hypothesize that the impact of any significant dizziness, including BPPV-related RD in children, would be similar. However, to the best of our knowledge, no BPPV-related RD has been reported in pediatric studies.

## 4 Risk factors for BPPV-related RD

Several risk factors have been proposed for BPPV-related RD symptoms (1). A meta-analysis of 4,487 patients from 31 studies identified age, female sex, secondary BPPV, a longer duration of BPPV before treatment, abnormal ocular vestibular evoked myogenic potential (oVEMP), abnormal cervical vestibular evoked myogenic potential (cVEMP), a higher Dizziness Handicap Index (DHI) score before treatment, anxiety, osteopenia, onset in winter, and a history of BPPV as risk factors for BPPV-related RD in patients with BPPV after successful repositioning. The following did not correlate with the occurrence of BPPV-related RD: the affected side, location or type of semicircular involvement, hyperlipidaemia, diabetes, hypertension, heart disease, migraine, sleep disorders, canalolithiasis/cupulolithiasis, the number of times a CRM was performed, and the number of vertigo attacks (9). A correlation between BPPV-related RD and age has also been reported in studies not included in the meta-analysis (18, 19). Although 12 studies in the meta-analysis by Ke et al. (9) reported that the number of CRMs may be a risk factor, the classification of the number of CRMs varied between studies, and the authors concluded that the reliability of the results was questionable. Other studies have reported that hypertension and diabetes are risk factors (20, 21). In addition, despite a high level of interest in the potential for vitamin D deficiency to be a risk factor for BPPV-related RD, only one study has reported a potential association between low levels of 25(OH)D in female patients aged < 50 years and an increased risk of moderate-to-severe BPPV-related RD 1 week after successful CRMs (22).

With regard to the relationship between oVEMPs and cVEMPs, there is currently no indisputable evidence in humans that VEMPs can provide specific information on otolith function. Some studies in patients with BPPV have noted abnormal cVEMP and oVEMP values compared with healthy controls (23, 24). One study suggested that most changes were noted in oVEMPs, suggesting that utricular dysfunction may be more common (especially in the earlier stages) than saccular dysfunction, although the entire vestibule was affected (24). Furthermore, patients with greater utricle and superior vestibular nerve involvement, as measured by oVEMP, showed delayed clinical recovery, suggesting a possible prognostic role of the test (24). In contrast, studies in patients with BPPV-related RD have suggested that the absence of cVEMP could increase the risk of BPPV-related RD (25, 26). Nevertheless, despite the debate regarding the contribution of non-otolith afferents to VEMPs, it is agreed that VEMPs originate from the transient otolith system and not from the sustained otolith system. BPPV-related RD is more likely to be caused by dysfunction in the sustained system, thereby making the use of VEMPs less useful (27). Overall, more research is needed to fully understand the implications of normal or abnormal VEMP responses in BPPV- and BPPV-related RD, and it should be noted that abnormal responses in any other vestibular test might be correlated with BPPV-related RD.

It is clear from the wide range of reported incidence rates and risk factors that BPPV-related RD is a heterogeneous condition with several underlying etiologies. It has been suggested that there may be no explanation for the cause of BPPV-related RD in >50% of patients (28). It is also likely that BPPV-related RD may differ between populations. For example, it has been reported that Asian people are 2.6 times more likely to present with BPPV compared with their Caucasian counterparts (29), possibly related to the higher prevalence of diabetes mellitus and its complications in the Asian population (30).

The recurrence of BPPV shares many of the same risk factors with BPPV-related RD. A recent meta-analysis of 14 predominantly Asian studies involving 3,060 BPPV patients reported that BPPV recurrence was closely related to female sex, hypertension, diabetes mellitus, hyperlipidaemia, osteoporosis, and vitamin D deficiency (31). This supports an earlier study of >1,000 BPPV patients in 11 centers across seven countries, in which ≥1 and ≥2 comorbid disorders (hypertension, diabetes, osteoarthrosis, osteoporosis, or depression) were found in 20 and 37% of subjects with recurrent BPPV, respectively (32). A reduction in bone mineral density has been reported to be independently associated with BPPV-related RD (33). More large-scale prospective studies in different countries are required to further investigate the risk factors for BPPV-related RD and how they might differ based on ethnicity.

## 5 Proposed mechanisms for BPPV-related RD

**Figure 3:**
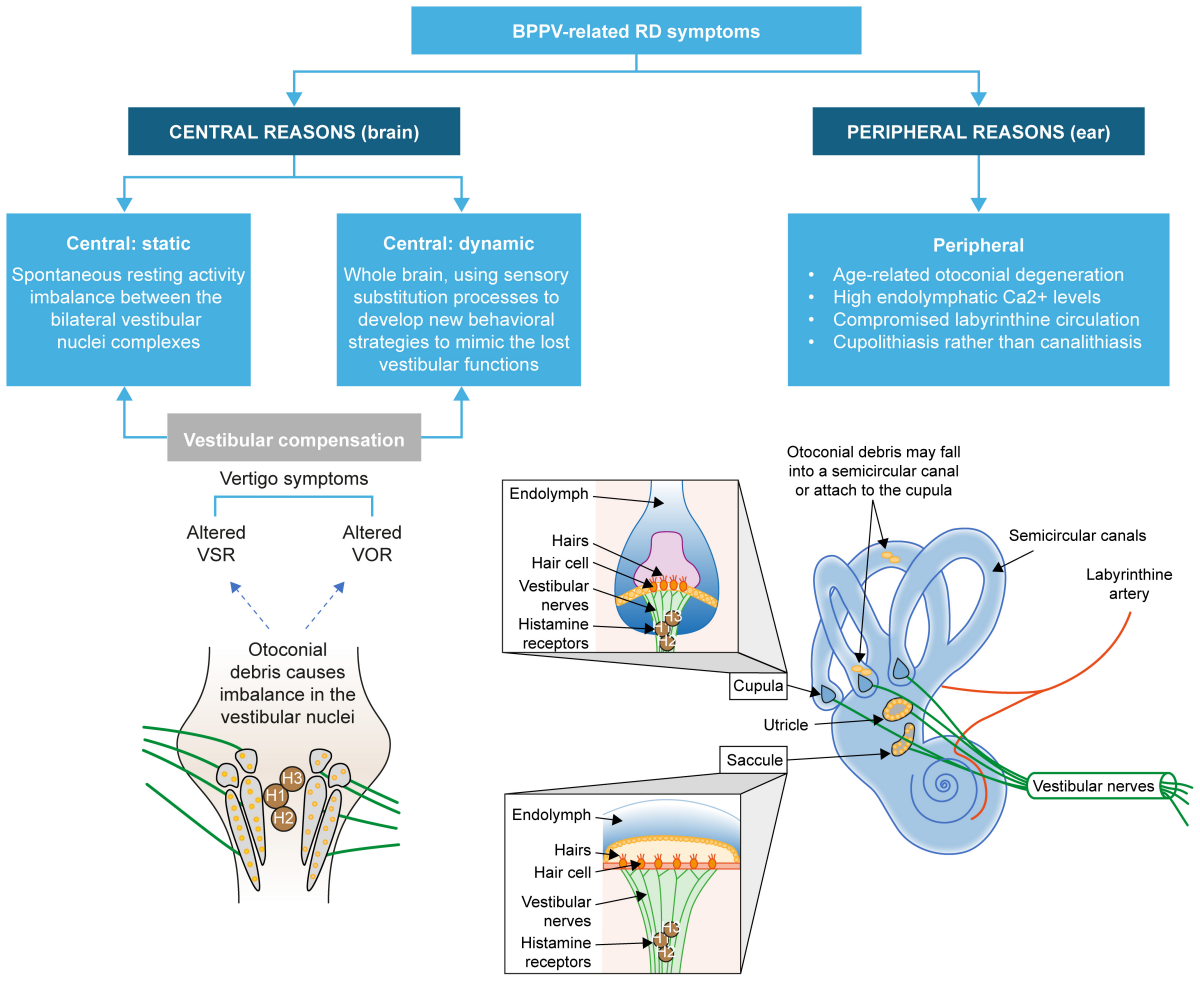
Peripheral and central causes of residual dizziness. Adapted from Özgirgin ON, et al. Corrigendum: Residual dizziness after BPPV management: exploring pathophysiology and treatment beyond canalith repositioning maneuvers. *Front Neurol*. 2024 Jul 29;15:1461600. doi: 10.3389/fneur.2024.1461600 Erratum for: *Front Neurol*. 2024 May 24;15:1382196. doi: 10.3389/fneur.2024.1382196 (1). BPPV, benign paroxysmal positional vertigo; RD, residual dizziness; VOR, vestibular ocular reflex; VSR, vestibular spinal reflex.

The proposed mechanisms for the development of BPPV-related RD are related to effects in the peripheral and central vestibular systems (1) (Figure 3). These mechanisms are not mutually exclusive, and their relative contributions may explain the reported differences in the risks and/or incidence of BPPV-related RD and relate to the duration and/or severity of symptoms. Understanding their roles and identifying them in patients may help guide clinicians in the management of patients.

### 5.1 Peripheral causes of BPPV-related residual dizziness

#### 5.1.1 Persistence of otoconial debris

Incomplete repositioning during CRM can result in residual otoconial debris that is sufficient to cause mild positional vertigo or less specific chronic or positional dizziness but insufficient to deflect the cupula to the degree required to provoke overt nystagmus (1). Furthermore, although CRM may clear debris from the canal, it may not completely resolve the utricular pathophysiology and, therefore, contribute to or cause BPPV-related RD.

#### 5.1.2 Effects of aging

The incidence of both BPPV and BPPV-related RD increases with age, thought to be due, in part, to age-related otoconial degeneration and vestibular hair cell loss (34). Animal studies have provided evidence for a relationship between osteopenia and changes in otoconial structure (35), which is supported by several clinical studies on BPPV (2). For example, among 32 women aged 50–85 years with idiopathic BPPV, 75% had osteopenia/osteoporosis on dual X-ray absorptiometry (36). A seasonal incidence of BPPV (37, 38) and BPPV-related RD has also been reported (9), which may be related to the association between winter months and low vitamin D levels and subsequently on bone mass through effects on calcium and bone metabolism (39, 40). Older adults are already at risk of vitamin D insufficiency due to decreased cutaneous synthesis and dietary intake (41). In addition, cupulolithiasis, in which free-floating endolymph debris adheres to the cupular membrane and renders the canal responsive to gravity, appears to be more likely in older patients and is more resistant to effective treatment with CRMs (1, 42).

#### 5.1.3 Endolymph calcium levels

Animal studies have shown that otoconia can dissolve in the endolymph within approximately 20 h; however, high endolymphatic Ca^2+^ levels slow the time of otoconia dissolution considerably (2, 43). Therefore, it is possible that a critical mass of otolith debris may accumulate in patients with high endolymphatic Ca^2+^ levels and cause BPPV-related RD symptoms (1). The biologically active form of vitamin D is involved in the upregulation of epithelial Ca^2+^ channel transporters that maintain low endolymph Ca^2+^ concentrations in the inner ear (22).

#### 5.1.4 Microcirculatory dysfunction

Blood vessels inside the cochlea have no collateral circulation. Otolith detachment in idiopathic BPPV may be secondary to ischaemia of the neuroepithelium of the utricular macula or semicircular canals (1). A combination of hypertension and diabetes may lead to tissue hypoxia and cochleovestibular degeneration (44). Hyperinsulinism may disrupt inner ear haemostasis and alter the ionic and metabolic characteristics of the stria vascularis, while hyperglycaemia increases vascular resistance by inhibiting nitric oxide-related vasodilation (45). Arterial plaques may trigger intravascular thrombosis and cause hypoperfusion of the inner circulation (45). Comorbid type 2 diabetes is associated with an increase in BPPV rates, often mediated by concurrent hypertension (29), which may be undiagnosed in around one-third of patients presenting with any type of vertigo (46). The incidence of hypertension and orthostatic hypotension in patients with BPPV-related RD has been reported as significantly higher than that in patients without BPPV-related RD (6, 47). Patients with BPPV and a combination of cardiovascular comorbidities, especially elderly patients with white matter hyperintensity, an indicator of chronic microvascular ischaemia, are more likely to develop BPPV-related RD (48). In a study of 149 patients with BPPV-related RD, 78 did not resolve spontaneously; most had high blood pressure and/or diabetes, 47 had hyperlipidaemia, 110 had heart disease, and 43 had ischaemic encephalopathy (21). Epidemiological evidence has also indicated an association between low vitamin D levels and diseases that affect microcirculation, such as hypertension (49) and diabetes (41, 50).

#### 5.1.5 Insufficient/inadequate CRMs

The outcome of CRM is dependent on the type and precise execution of the maneuver, the latter being affected by the angle and angular velocity of the head and the duration of each step, fluid dynamics, and the amount, size, and location of otoconia in the canal (1). CRMs may be less effective in patients with secondary BPPV or lateral canal cupulolithiasis, with the latter being more common than other types of BPPV in older patients (1, 51).

The clinical relevance of the various proposed risk factors is summarized in Table 1, which includes evaluation of the evidence available for BPPV-related RD, which is largely variable and based on small studies and the authors' combined clinical experience.

**Table 1:** CLEAR risk factor checklist for peripheral causes of BPPV-related RD.

**Peripheral risk factor**	**Mechanism**	**Impact score**
Previous BPPV episodes (9)	More episodes may indicate persistent otolith degeneration and/or the presence of comorbid conditions that increase the risk of RD^*^	**2**
History of recurrent BPPV (>2 episodes per year) (9)	Suggests ongoing otolith instability and/or the presence of comorbid conditions that increase the risk of RD^*^	**3**
Vitamin D deficiency (9)	The biologically active form of vitamin D is involved in the upregulation of epithelial Ca^2+^ channel transporters that help maintain low endolymph Ca^2+^, retain the capacity to dissolve exfoliated otoconia, and prevent abnormal otoconia (1, 22) Decreased serum levels of 25(OH)D were associated with recurrence of BPPV in a Chinese population (52) Vitamin D serum level is lower in canalolithiasis vs. cupulolithiasis of the horizontal canal (53) Epidemiological evidence indicates an association between low vitamin D and diseases that affect the microcirculation (41)	**2**
Osteopenia (9, 54)	Calcium metabolism abnormalities could affect otolith regeneration and vestibular compensation (1)	**2**
Hearing loss or cochlear damage	Experiencing both hearing loss and RD suggests an inner ear problem, most commonly Ménière's disease^*^ Complications post-cochlear implant may cause dizziness (55)	**1**
Female sex (9, 56)	Otoconia may be more prone to degeneration and detachment in women (56, 57) Females are more likely than males to be affected by vitamin D deficiency (53, 58) Fluctuations in estrogen levels can lead to increased vestibular dysfunction (59)	**2**
**Peripheral risk factors specific to BPPV diagnosis**		
BPPV not restricted to one canal (9)	Bilateral BPPV is associated with greater impairment of vestibular function^*^ Multi-canal BPPV involvement is associated with resistance to treatment with standard CRMs (60)	**2**
Severe vertigo before CRM	Indicates greater inner ear disturbance^*^	**2**
Multiple maneuvers required for resolution (20)	Suggests more severe dysfunction^*^	**3**
**Peripheral risk factors due to systemic and metabolic factors**		
Diabetes or metabolic syndrome (20)	Affects microcirculation and may delay otolith recovery (29)	**2**
Hypertension (20)	May affect inner ear microcirculation, potentially leading to hypoxia and cochleovestibular degeneration (46)	**3**

The proposed risk level is based on the combination of published evidence (cited where available) and clinical experience^*^: 1 (yellow), low risk of developing BPPV-related RD; 2 (amber), medium risk of developing BPPV-related RD; 3 (red), high risk of developing BPPV-related RD.

BPPV, benign paroxysmal positional vertigo; CRM, canalith repositioning maneuver; RD, residual dizziness.

### 5.2 Central causes of BPPV-related residual dizziness

#### 5.2.1 Incomplete vestibular compensation

**Figure 4:**
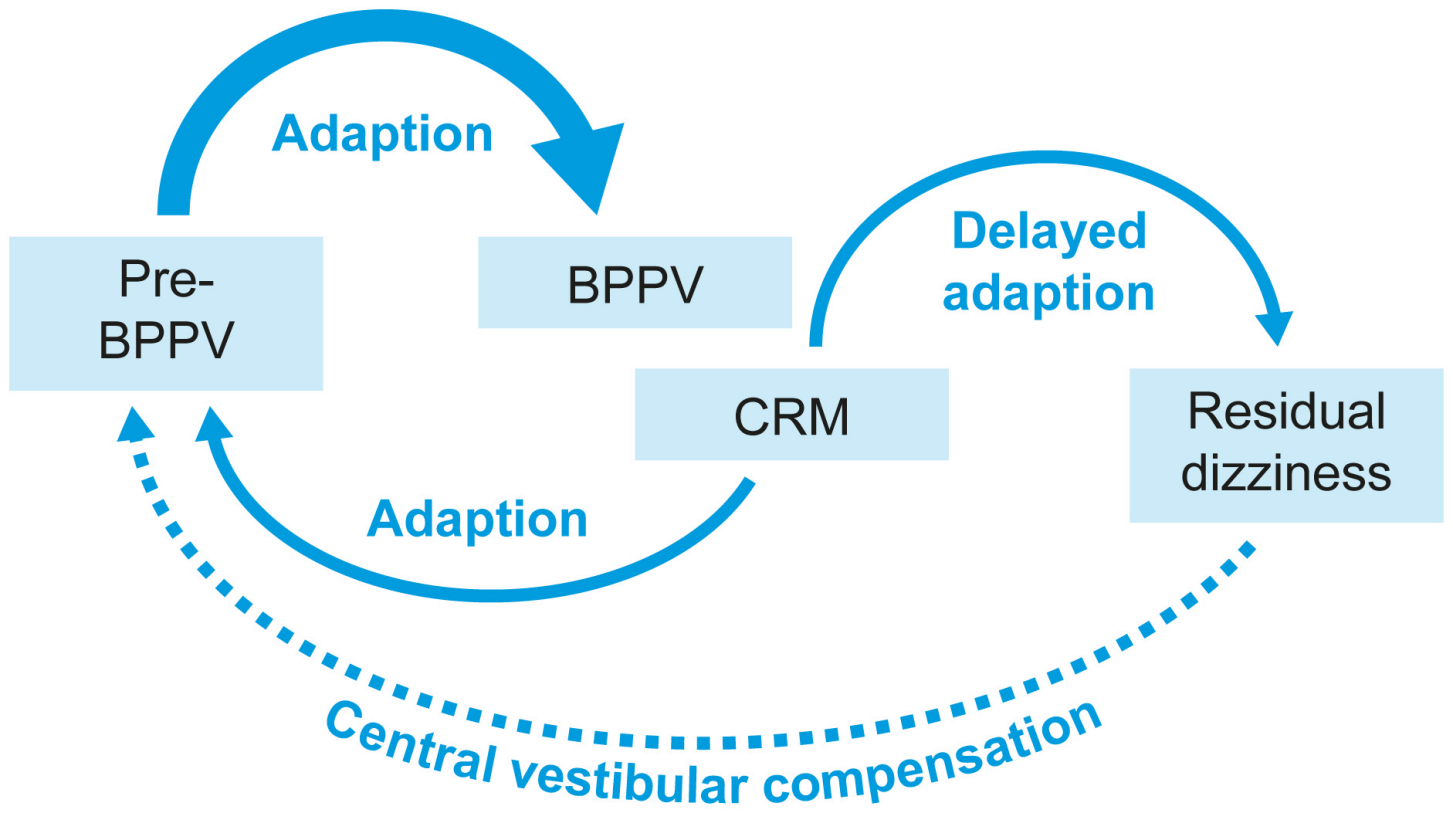
A visual representation of how delayed adaptation might cause BPPV-related RD symptoms. BPPV, benign paroxysmal positional vertigo; CRM, canalith repositioning maneuver; RD, residual dizziness.

The static components of dizziness after vestibular loss (those that occur when the head is kept still) are due to the spontaneous resting activity imbalance between the bilateral vestibular nuclei complexes (61, 62). Complete recovery of vestibular symptoms requires adequate vestibular compensation in the central nervous system (63). Incomplete vestibular compensation may be associated with some of the same factors that increase the risk of BPPV-related RD, such as the duration of BPPV, the patient's emotional state, or the inability of the central nervous system to quickly readjust to a new functional status (64, 65). It has been proposed that the persistence of otoconial debris may alter the tonic discharge from the affected labyrinth, causing a functional asymmetry that induces a new adaptation due to rebalancing of the activity between the vestibular nuclei (4, 66). This new adaptation becomes more established the longer the otoconial debris remains in the endolymph, hence the association between the duration of BPPV and BPPV-related RD. While the brain is able to adapt quickly in the first instance, BPPV-related RD symptoms may occur when the brain takes an extended time to readapt to the old pattern after resolution of BPPV due to successful CRM(s) (66) (Figure 4). Dynamic deficits (those that occur when the head is moving) may be poorly compensated and exhibited over a longer time (67), as they are related to whole-brain activity involved in developing sensory substitution processes to develop new behavioral strategies that mimic lost vestibular functions.

#### 5.2.2 Duration of BPPV

**Figure 5:**
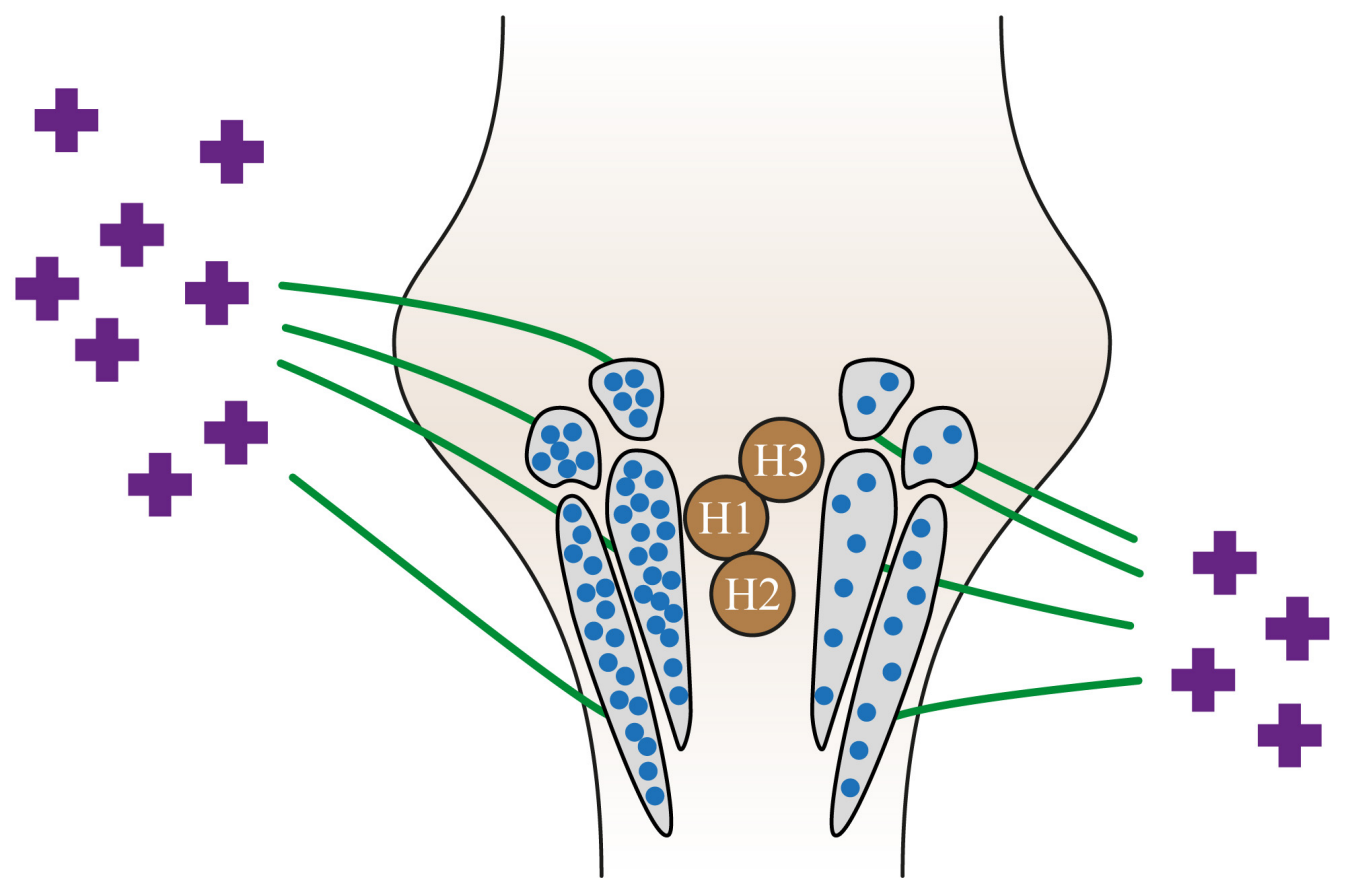
A visual representation of how altered tonic discharge from the affected labyrinth affects the vestibular nuclei. Adapted from Özgirgin ON, et al. Corrigendum: Residual dizziness after BPPV management: exploring pathophysiology and treatment beyond canalith repositioning maneuvers. *Front Neurol*. 2024 Jul 29;15:1461600. doi: 10.3389/fneur.2024.1461600 Erratum for: *Front Neurol*. 2024 May 24;15:1382196. doi: 10.3389/fneur.2024.1382196 (1). H1, H2, and H3 are the histamine receptors in the vestibular nuclei.

The persistence of debris in the semicircular canal after CRM can alter tonic discharge from the affected labyrinth. Such functional asymmetry (Figure 5) can induce a new adaptation through rebalancing of the activity between the vestibular nuclei, and this new condition tends to neutralize the imbalance produced in the peripheral vestibular system (4, 5, 66). A small study reported that patients with a long duration of BPPV were more likely to have moderate-to-severe BPPV-related RD after successful CRM and that the RD impacted mainly the areas of social function and emotional psychology (67). As the rate of undiagnosed BPPV is high at all ages (2), many patients might have BPPV that has already existed for a long time at diagnosis, potentially putting them at a high risk of RD. Although most reports of BPPV-related RD are in adults, long delays in the diagnosis of BPPV are common in children (mean 172.2 days), reflecting a lack of awareness about BPPV among pediatric healthcare providers (68), and also a potential cause of BPPV-related RD, although this does appear to be rare in children.

#### 5.2.3 Comorbid vestibular conditions

The risk of BPPV-related RD in secondary BPPV is 1.88 times that of idiopathic BPPV, with Ménière's disease and vestibular neuritis being two of the most common causes of secondary BPPV, along with head trauma (9). This is supported by a retrospective analysis of data from the US Vestibular Disorders Association Registry reporting that 83% of adults with BPPV had received an additional vestibular diagnosis or may have had atypical BPPV (69). Patients with BPPV and other concomitant vestibular pathologies have been reported to have a proportionally higher persistence of symptoms after CRMs than patients with pure BPPV (70).

#### 5.2.4 Anxiety and/or depression

The high comorbidity between balance disorders and anxiety is a complex, two-way interaction whereby patients with psychological symptoms commonly report vestibular symptoms, such as dizziness, and conversely, patients with vestibular dysfunction report psychological symptoms (71, 72). Overlapping neural circuits between anxiety and the balance control system may provoke increased anxiety levels in patients after BPPV, and patients who develop higher anxiety have incomplete central adaptation (72). There is evidence that dopamine plays an important role in anxiety modulation in different parts of the brain, and disruption of dopaminergic efferent pathways could be associated with vestibular dysfunction (73, 74). Dopamine has been shown to improve vestibular compensation in aged rats, likely via a mechanism involving vestibular nuclei (74). Varying expression of dopamine has been reported in the subepithelial stroma of the saccule, along with intense expression of the H3 receptor in epithelial lining cells of the saccule (75); H3 agonism plays a role in reducing dopaminergic signaling (76).

Based on the evaluation of the evidence available and combined clinical experience, the impact of the central risk factors is summarized in Table 2.

**Table 2:** CLEAR risk factor checklist for central causes of BPPV-related RD.

**Central risk factor**	**Mechanism**	**Impact score**
Age >65 years (9, 19, 20, 28)	Slower central compensation (34)	**2**
History of vestibular migraine (1)	Central vestibular dysfunction delays compensation (77, 78)	**3**
Cognitive impairment (79, 80)	Slows adaptation to new vestibular input (81), especially in the elderly	**2**
Female sex (9, 56)	Fluctuations in estrogen levels can lead to impaired compensation after BPPV treatment (82) Changes to hormone receptor stimulation may be central to the pathogenesis of BPPV (57, 59, 83)	**2**
Physical inactivity (66)	Reduces vestibular compensation efficiency (84)	**1**
Longer duration of BPPV (9, 85) Symptoms >4 weeks before CRM^*^	Prolonged compensation period (64, 66)	**2**
Severe oscillopsia during a BPPV episode	Indicates prolonged adaptation difficulty, especially when combined with VM comorbidity^*^	**3**
Residual mild dizziness post-maneuver (9)	Suggests incomplete adaptation (4, 64)	**3**
Persistent non-typical BPPV nystagmus post-maneuver	Could indicate residual debris or incomplete adaptation^*^	**1**
Anxiety or depression (9, 20, 80)	Increases hypervigilance, prolongs symptom perception (86, 87) Anxiety disorders are linked to chronic dizziness conditions, such as persistent postural-perceptual dizziness (88)	**2**
Chronic stress or PTSD	Prolongs hypervigilance and dizziness perception^*^	**2**

The proposed risk level is based on the combination of published evidence (cited where available) and clinical experience^*^: 1 (yellow), low risk of developing BPPV-related RD; 2 (amber), medium risk of developing BPPV-related RD; 3 (red), high risk of developing BPPV-related RD.

BPPV, benign paroxysmal positional vertigo; CRM, canalith repositioning maneuver; PTSD, post-traumatic stress disorder; RD, residual dizziness; VM, vestibular migraine.

## 6 Important differential diagnoses

If a patient presents with RD after successful CRM for BPPV or after suspected spontaneous resolution of BPPV, several key differential diagnoses should be considered in addition to BPPV-related RD. These include vestibular migraine and Ménière's disease, as well as other central or peripheral causes of dizziness. The diagnostic criteria for vestibular migraine are ≥5 episodes of vestibular symptoms of moderate-to-severe intensity, lasting between 5 min and 72 h; at least 50% of these episodes accompanied by ≥1 migraine symptom(s), including headache (with ≥2 features of unilateral, throbbing, moderate/severe intensity, aggravated by routine physical activity), photophobia and phonophobia, or visual aura; current or previous history of migraine with or without aura; and not better accounted for by another diagnosis (89). Several vestibular symptoms may be reported in vestibular migraine, including spontaneous and triggered vertigo/dizziness (90). A confirmed diagnosis of Ménière's disease is assumed with ≥2 episodes of spontaneous vertigo, each lasting 20 min to 12 h with fluctuating aural symptoms (hearing changes, tinnitus, and aural fullness) before, during, or after an episode of vertigo; audiometrically documented low-to-medium frequency sensorineural hearing loss in one ear on at least one occasion, starting before, during, or after an episode of vertigo; and not better explained by another vestibular diagnosis (91). Key differential diagnoses of central origin include stroke and other neurological findings such as a tumor or vertebrobasilar insufficiency. Key differential diagnoses of peripheral origin include anxiety, concomitant medications, cardiometabolic conditions, and orthostatic hypertension.

## 7 Introducing the concept of type 1 and type 2 BPPV-related RD

Based on the authors' combined clinical experience of over 90 years in treating thousands of patients with vestibular disorders, we propose that there are two types of BPPV-related RD, differentiated by their initial presentation (Table 3). Type 1 BPPV-related RD is defined as an initial presentation with a typical BPPV nystagmus that is resolved with ≥1 CRM, with RD symptoms reported at the follow-up appointment (at 1–2 weeks post-CRM). However, we recognize that some patients present with dizziness symptoms without positional nystagmus but with a clear history of BPPV, which was not examined or diagnosed and has apparently resolved spontaneously, and we propose that this type of RD can be defined as type 2 BPPV-related RD. Current evidence indicates that BPPV may resolve spontaneously without intervention in approximately 35%−50% of patients (92). The Consensus Document of the Committee for the Classification of Vestibular Disorders of the Bárány Society designates this presentation as “probable BPPVo, spontaneously resolved” (pBPPVsr). This entity is defined as recurrent, brief (< 1 min) episodes of positional vertigo or dizziness precipitated by lying down or turning over in the supine position, in the absence of observable nystagmus or vertigo during positional maneuvers, and not attributable to any alternative disorder (93). With careful questioning to obtain a detailed clinical and symptom history, we can determine whether there was indeed a BPPV that spontaneously resolved. As has been previously reported, patients' descriptions of symptoms are not reliable (94), and they may find it difficult to differentiate between vertigo and dizziness. However, patient reports of timing and triggers have been found to be reliable (94). An experienced neurologist/ear, nose, and throat (ENT) specialist can identify cases of spontaneously resolved BPPV from the characteristic timing and triggers associated with BPPV and, after confirming the absence of suggestive nystagmus and excluding other potential causes, can then reliably recognize BPPV-related RD (Table 4).

**Table 3:** A new concept for the identification of BPPV-related RD based on initial presentation.

**Type 1 BPPV-related RD**	**Type 2 BPPV-related RD**
• Initial presentation: vertigo with a typical BPPV nystagmus • Treatment: ≥1 CRM to resolve nystagmus • At follow-up: RD symptoms	• Initial presentation: dizziness symptoms but absence of a typical BPPV nystagmus • Clinical history: symptoms that allow retrospective diagnosis of BPPV with a high level of certainty by an experienced neurologist/ENT/otologist

BPPV, benign paroxysmal positional vertigo; CRM, canalith repositioning maneuver; ENT, ear, nose, and throat specialist; RD, residual dizziness.

**Table 4:** Key clinical features of BPPV that have resolved spontaneously.

**Feature**	**Features that are suggestive of BPPV**
Onset	Sudden or after a provocative movement, e.g., getting up too fast, after a head bump, viral illness, or prolonged bed rest
Time of onset	There is no minimum/maximum timing between the event and the presentation
Trigger	Vertigo triggered positionally, e.g., rolling over in bed, lying down, sitting up, bending forward, or looking up (“top shelf vertigo”)
Type of dizziness	Classic spinning sensation (true vertigo), often described as “the room spinning”
Duration of attacks	Short-lived (typically < 30–60 s), with abrupt start and stop
Latency	A few seconds delay (1–5 s) between head movement and symptom onset
Associated symptoms	Nausea may occur, but no hearing loss, tinnitus, diplopia, or limb symptoms
Frequency	Episodes often cluster, then spontaneously remit; recurrences are common
Pattern of recovery	Vertigo subsides when the patient avoids provocative movements

BPPV, benign paroxysmal positional vertigo.

While the management options are the same for both type 1 and type 2 BPPV-related RD, it is an important distinction to ensure that we are capturing all cases of dizziness that are related to BPPV.

## 8 Follow-up strategies for BPPV-related RD

Once identified as type 1 or type 2 BPPV-related RD, there are several potential follow-up strategies. The first is the avoidance of pharmacological agents that act as vestibular suppressants and interfere with central compensation (96). It may seem attractive to use medication to suppress dizziness and other symptoms, but clinicians should be aware that they may interfere with central compensation (97) and make the symptoms worse. Clinicians should consider the use of techniques and/or medications that facilitate central compensation. Evidence suggests that patients with high anxiety benefit less from vestibular rehabilitation (VR) (97) and have a greater impact on their quality of life than those with lower anxiety (81). Identifying such patients could be important when considering management options. Although the DHI and visual analog scale (VAS) are the most commonly used measures to quantify RD, neither of these has been validated specifically for BPPV or RD. The DHI is a self-assessment inventory of 25 items covering the functional, emotional, and physical impacts on daily life, whereas the VAS has the advantage of being able to distinguish dizziness from vertigo, with reliable results in evaluating RD (1).

For BPPV, there is evidence that CRMs are more effective in the short term than exercise-based vestibular rehabilitation (VR), although a combination of the two is effective for long-term functional recovery (98). However, there is insufficient evidence and no consensus on the most effective form of VR (99, 100), likely due to differences in endpoints, use of pharmacological options, and patients' ability to complete VR as instructed. Although some studies have shown that VR can effectively relieve or reduce residual symptoms in patients with BPPV (101, 102), others have shown no benefit (103).

Important comorbidities should also be identified and treated appropriately to reduce the risk of recurrence (Table 5).

**Table 5:** Potential management options for BPPV-related RD.

**Intervention**	**Mechanism / Rationale**	**Clinical Notes / Evidence**
**A. Avoid vestibular suppressants**		
Pharmacotherapy • Avoid benzodiazepines (e.g., diazepam, clonazepam)	Inhibitory effect on the electrical activity of the vestibular nuclei (104) and central compensation (95)	Benzodiazepines (BZDs) may interfere with central compensation and are not recommended for routine use in patients with BPPV • Current guidelines recommend against the routine treatment of patients with BPPV with vestibular suppressant medications such as BZDs (95) • BZDs, such as diazepam and clonazepam, have anxiolytic, sedative, muscle-relaxant, and anticonvulsant properties (95) • In prolonged dizziness, BZDs can reduce the subjective sensation of spinning but can also interfere with central compensation (95, 105) • Although BZDs may provide initial relief for acute dizziness symptoms, routine use is not recommended due to the risk of dependence, cognitive impairment, falls, and physical injury (105, 106) • In patients with BPPV, BZDs may have no effect on symptom resolution at the point of longest follow-up (107) • In the occasional patient who cannot tolerate a CRM, is exceptionally anxious about undergoing a CRM, or has RD after a CRM, it may be appropriate to treat for 2–3 days with a vestibular suppressant (107)
Pharmacotherapy • Avoid antihistamines (e.g., diphenhydramine, dimenhydrinate, cinnarizine, meclizine, promethazine)	Interferes with central compensation after vestibular injury (95)	Antihistamines may interfere with central compensation and are not recommended for routine use in patients with BPPV • Current guidelines recommend against the routine treatment of patients with BPPV with vestibular suppressant medications such as antihistamines (95) • Antihistamines appear to have a suppressive effect on the central emetic center to relieve the nausea and vomiting associated with motion sickness, for example, meclizine, diphenhydramine, and promethazine (a phenothiazine with antihistamine properties), but interfere with central compensation (95, 105) • Vestibular suppressant antihistamines have the potential for significant harm, such as drowsiness, cognitive deficits, and interference with driving or operating machinery (95) • Vestibular suppressants such as antihistamines are not routinely recommended for treatment of BPPV, other than for the short-term ( ≤ 3 days) management of autonomic symptoms, such as nausea or vomiting, in a severely symptomatic patient (95, 106, 107); in patients with BPPV, pheniramine, cinnarizine, and flunarizine may have no effect on symptom resolution at the point of longest follow up (107)
Pharmacotherapy • Avoid phenothiazines (i.e., prochlorperazine)	Suppresses central vestibular nuclei and pathways (108)	Current guidelines recommend against the routine treatment of patients with BPPV with vestibular suppressants (95) • Vestibular suppressants have the potential for significant harm, such as drowsiness, cognitive deficits, and interference with driving or operating machinery (95) • Vestibular suppressants are not routinely recommended for treatment of BPPV, other than for the short-term ( ≤ 3 days) management of autonomic symptoms, such as nausea or vomiting, in a severely symptomatic patient (95, 106)
**B. Facilitate central vestibular compensation**		
Vestibular rehabilitation therapy (VRT; e.g., customized balance, gaze, and habituation exercises)	The goal of VRT is to enhance vestibular compensation and neuroplasticity by focusing on gaze stabilization, postural control, and sensory integration (109) to leverage multisensory input (visual, proprioceptive, and vestibular), recalibrate postural control, and reduce dizziness (110)	VRT improves RD by promoting central compensation • VRT has been shown to significantly reduce the severity and duration of BPPV-related RD, especially when individualized to patient deficits (95) • Individualized programmes can reduce RD severity (110) • Treatment typically lasts 4–8 weeks, and while home programmes exist, clinician guidance is preferred to optimize outcomes (95)
Encourage physical activity / mobility	• May enhance neuroplasticity phenomena, making the vestibular system more receptive to natural compensatory mechanisms (111) • Physical activity supports vestibular compensation through adaptive plasticity, reduces anxiety, and prevents deconditioning (112)	Daily activity supports vestibular recovery and may reduce RD duration • Daily activity supports vestibular recovery (111) and may reduce RD duration, regardless of age (66) • Regardless of age, the resumption of regular daily physical activities is associated with a lack of RD after BPPV (66) • Unless contraindicated by fall risk or severe instability, patients should be encouraged to maintain daily physical activity throughout the recovery phase
Pharmacotherapy • Agents that facilitate central vestibular compensation (e.g., betahistine)	• Betahistine may promote and facilitate central vestibular compensation, enhancing histamine synthesis within tuberomammillary nuclei of the posterior hypothalamus and histamine release within vestibular nuclei through antagonism of H3 autoreceptors, and increasing alertness regulation through cerebral H1 receptors (96, 113) • Additionally, betahistine may increase vestibulocochlear blood flow, supporting inner ear perfusion (113)	Betahistine 48 mg/day for ~3 months may improve RD by promoting central compensation • There is robust strong evidence from animal studies to demonstrate the effect of betahistine on central compensation (1, 96) • In humans, the bioavailability of oral betahistine is reduced by first-pass metabolism in some patients (96). Studies are ongoing to identify strategies to overcome the first-pass effect and to identify optimal doses to support central compensation (96, 114–117) • As the administration of betahistine is dose-dependent and time-dependent, selecting the appropriate dose and duration to achieve desirable effects is important (117) • Treatment with betahistine 48 mg/d for up to 90 days was also useful to manage the nausea and vomiting that may occur during CRMs and in treating dizziness that may continue after CRM (103). Betahistine 48 mg/d in addition to CRM in patients with PV may improve symptoms (118), normalize postural stability more rapidly (119) and speed recovery rate (120) compared with CRM alone • Combining betahistine with CRM could improve the outcomes of *BPPV* in the long term, according to two meta-analyses (121, 122)
**C. Manage comorbidities**		
Vitamin D supplementation (1,000–2,000 IU/day)	Vitamin D plays a critical role in calcium regulation and otoconial integrity, which are essential for vestibular function. Deficiency may destabilize otoconia and impair vestibular compensation, especially in patients with comorbid osteoporosis, diabetes, or hypertension (22, 123). Low vitamin D levels are also associated with microvascular dysfunction that could compromise inner ear perfusion (49, 50)	Vitamin D may help stabilize otoconia and reduce recurrence of BPPV • Serum concentrations of 25(OH)D of ≥20 ng/ml (≥50 nmol/L) are generally considered adequate for bone and overall health in healthy individuals (124) • Vitamin D supplementation, in the absence of calcium, may be beneficial for patients prone to recurrent BPPV episodes, particularly when serum vitamin D levels are suboptimal (125, 126)
Address systemic and psychiatric comorbidities (e.g., hypertension, diabetes mellitus, anxiety)	• Hyperglycaemia and endothelial dysfunction impair nitric oxide-mediated vasodilation, reducing blood flow to inner ear structures (45) • Uncontrolled hypertension can lead to inner ear haemorrhage or ischaemia that can compromise cochleovestibular function and/or affect central functions when areas of the brain involved in the vestibular network are involved (46) • Overlapping neural circuits between anxiety and the balance control system may provoke increased anxiety levels in patients after BPPV. Anxiety is associated with impaired central through disruption of vestibular-cortical integration, altering neuroplastic adaptation (5, 62, 72)	Optimizing factors that can impede vestibular compensation, such as cardiometabolic health and psychological stress, is an essential component of BPPV-related RD management • Identify and optimize management of hypertension and diabetes mellitus • Manage anxiety through education initially, with referral to counseling/psychiatrist as appropriate^*^ • Screening and referral for anxiety-related vestibular symptoms should be part of standard care, particularly in older adults, as recommended by the World Falls Guidelines (127)

BPPV, benign paroxysmal positional vertigo; BZD, benzodiazepine; CRM, canalith repositioning maneuver; RD, residual dizziness; VRT, vestibular rehabilitation therapy.

^*^Based on clinical experience.

It should be noted that while there is one study reporting the benefits of Danhong injection, a traditional Chinese medicine, in reducing BPPV-related RD (128), this has been omitted from Table 5 on the basis of insufficient evidence.

EGb 761, a special extract from dried leaves of *Ginkgo biloba*, has been approved for the treatment of vertigo of vascular and involutional origin in several countries (129). However, a recent meta-analysis of 25 randomized controlled studies with >1,200 patients concluded that the addition of *Ginkgo biloba* only provided clinical efficacy in patients with vertebrobasilar insufficiency, cervical vertigo, and non-disease-specific vertigo, but not in BPPV or Meniere's disease (130); for this reason, *Ginkgo biloba* has also not been included as an evidence-based management consideration.

## 9 The future of BPPV-related RD identification and management

Improved identification and management of BPPV-related RD could help prevent morbidity and loss of QOL. Especially in older patients, it is important to manage dizziness symptoms to avoid falls and diminished QOL, to identify any other vestibular or cardiovascular conditions, and to effectively differentiate BPPV-related RD from other sinister conditions that affect balance. In younger patients, the impact of BPPV-related RD is not well-understood; however, it might be expected to exert a substantial impact on work and activities of daily living. As the world population ages, BPPV-related RD is likely to become an increasingly problematic condition; increasing age causes age-associated degeneration of otoconia (2, 34) and an increased likelihood of vestibular and/or vascular conditions (72, 131).

Objective measures to assess utricular function in the absence of a BPPV-specific nystagmus have not been established in the context of BPPV-related RD, and while the Subjective Visual Vertical test and oVEMPs may identify utricular dysfunction, they have their limitations (1). Studies suggest that the video ocular counter-roll is simple to apply and has clinical value in identifying the stage of statolith function, loss, and recovery at the bedside; it consists of a simple maneuver during which the head and torso are tilted laterally en bloc with measurement of the torsional vestibulo-ocular reflex (132–134). The unilateral centrifugation test also measures utricular sensitivity and the preponderance of the right or left utricle. During this test, subjects are rotated about a vertical axis to align one utricle with the axis of rotation and then subjected only to gravitational forces to induce ocular counterrolling, which is measured online using three-dimensional video-oculography (135). While positive subjective visual vertical, oVEMP, unilateral centrifugation, and/or video ocular counter-roll results for utricular dysfunction are highly suggestive of BPPV-related RD, there is insufficient evidence to consider them conclusive of BPPV-related RD, and all require specialist equipment that may not be available for all patients.

**Figure 6:**
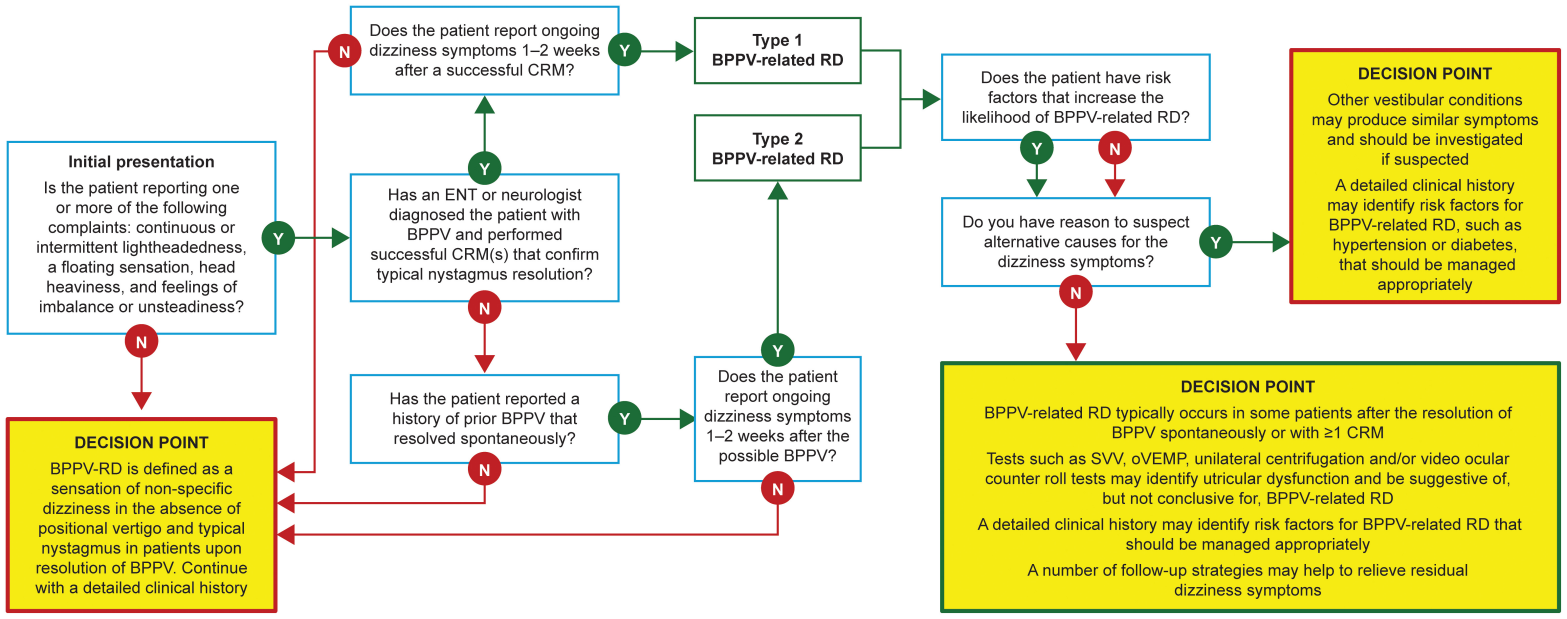
Overview of the CLEAR algorithm for use by specialist clinicians to support in the recognition of risk factors and follow-up strategies for BPPV-related RD. BPPV, benign paroxysmal positional vertigo; ENT, ear, nose, and throat specialist; CRM, canalith repositioning maneuver; oVEMP, ocular vestibular evoked myogenic potential; RD, residual dizziness; SVV, subjective visual vertical.

**Figure 7:**
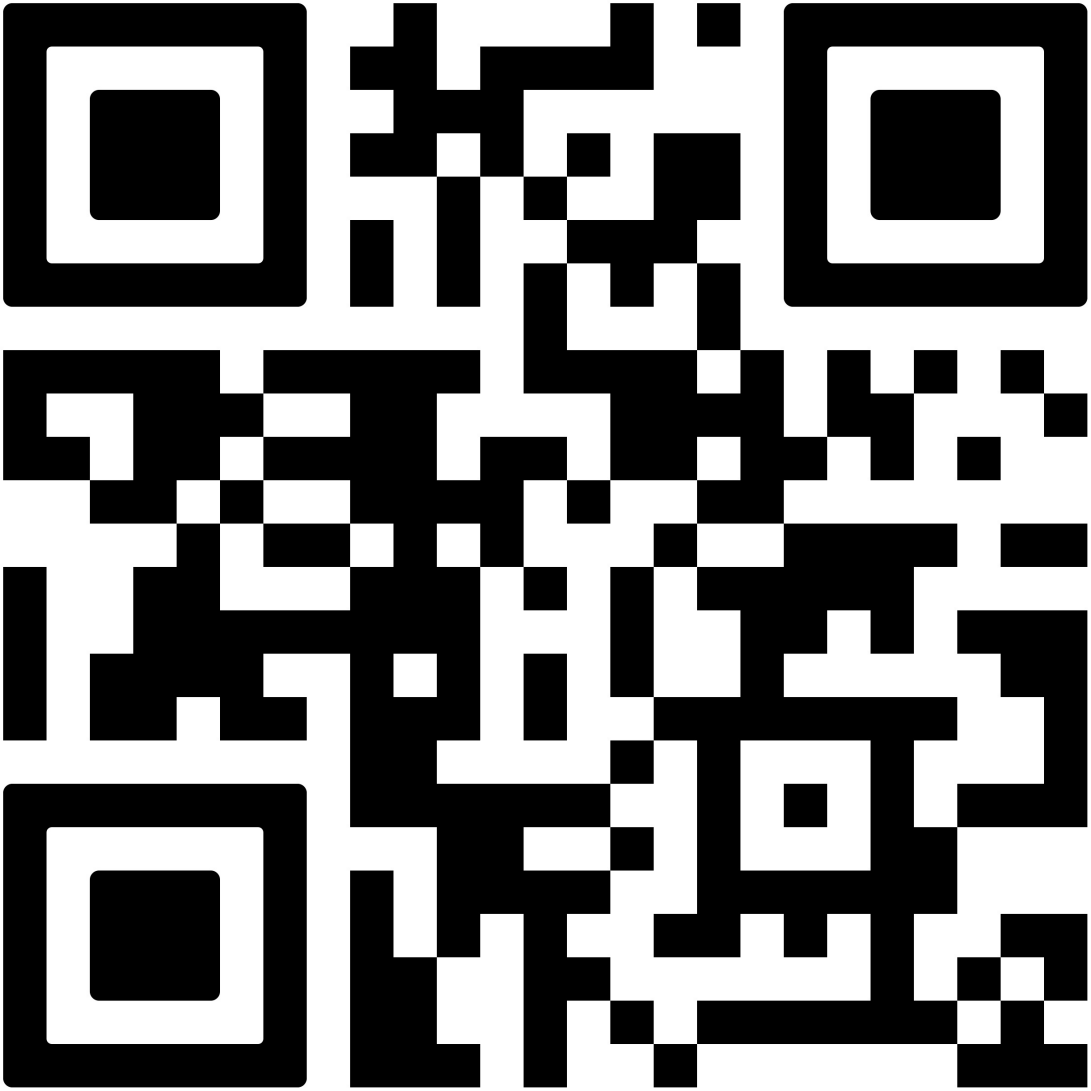
The Clinician-Led Evaluation for Assessment of Residual dizziness (CLEAR) algorithm.

Based on published evidence and our combined clinical experience, we have developed the Clinician-Led Evaluation for Assessment of Residual dizziness (CLEAR) tool, a BPPV-related RD algorithm to help specialist clinicians identify and manage patients (Figure 6; click on the QR code in Figure 7 to access). This algorithm is developed according to our understanding of the underlying causes and the applicability of potential treatment options. The tool is based on the data in Tables 1−4 and is available for use by specialists in interactive form via the QR code provided or https://clear-dizziness.org. As described earlier, Tables 1, 2 provide a list of the potential risk factors and their impact scores (mild, moderate, or severe) based on the combination of published evidence and the authors' clinical experience, which may assist your clinical decision-making by highlighting patients with a greater risk of BPPV-related RD. The CLEAR tool has been developed as a click-through algorithm (Figure 6) to help recognize patients with RD, with the option to download a summary (in PDF format) of the recommendations for follow-up strategies for each patient. Future research is needed to validate independent risk factors for BPPV-related RD, and we encourage the use of the algorithm in any such studies.

Ultimately, our aim is to help all healthcare professionals who work with patients with BPPV to promptly recognize BPPV-related RD, follow up appropriately to resolve symptoms as quickly as possible, and thereby improve patient outcomes.
